# Mitochondrial Dysfunction in Cancer and Neurodegenerative Diseases: Spotlight on Fatty Acid Oxidation and Lipoperoxidation Products

**DOI:** 10.3390/antiox5010007

**Published:** 2016-02-19

**Authors:** Giuseppina Barrera, Fabrizio Gentile, Stefania Pizzimenti, Rosa Angela Canuto, Martina Daga, Alessia Arcaro, Giovanni Paolo Cetrangolo, Alessio Lepore, Carlo Ferretti, Chiara Dianzani, Giuliana Muzio

**Affiliations:** 1 Dipartimento di Scienze Cliniche e Biologiche, Università di Torino, Torino 10125, Italy; stefania.pizzimenti@unito.it (S.P.); rosangela.canuto@unito.it (R.A.C.); martina.daga@unito.it (M.D.); giuliana.muzio@unito.it (G.M.); 2 Dipartimento di Medicina e Scienze della Salute “V. Tiberio”, Università del Molise, Campobasso 86100, Italy; gentilefabrizio@unimol.it (F.G.); alessia.arcaro@unimol.it (A.A.); giovanni.cetrangolo@unimol.it (G.P.C.); 3 Dipartimento di Medicina Molecolare e Biotecnologie Mediche, Università di Napoli Federico II, Napoli 80131, Italy; alessiolep@gmail.com; 4 Dipartimento di Scienze e Tecnologia del Farmaco, Università di Torino, Torino 10125, Italy; carlo.ferretti@unito.it (C.F.); chiara.dianzani@unito.it (C.D.)

**Keywords:** mitochondria, cancer, neurodegenerative diseases, fatty acid oxidation, lipoperoxidation products

## Abstract

In several human diseases, such as cancer and neurodegenerative diseases, the levels of reactive oxygen species (ROS), produced mainly by mitochondrial oxidative phosphorylation, is increased. In cancer cells, the increase of ROS production has been associated with mtDNA mutations that, in turn, seem to be functional in the alterations of the bioenergetics and the biosynthetic state of cancer cells. Moreover, ROS overproduction can enhance the peroxidation of fatty acids in mitochondrial membranes. In particular, the peroxidation of mitochondrial phospholipid cardiolipin leads to the formation of reactive aldehydes, such as 4-hydroxynonenal (HNE) and malondialdehyde (MDA), which are able to react with proteins and DNA. Covalent modifications of mitochondrial proteins by the products of lipid peroxidation (LPO) in the course of oxidative cell stress are involved in the mitochondrial dysfunctions observed in cancer and neurodegenerative diseases. Such modifications appear to affect negatively mitochondrial integrity and function, in particular energy metabolism, adenosine triphosphate (ATP) production, antioxidant defenses and stress responses. In neurodegenerative diseases, indirect confirmation for the pathogenetic relevance of LPO-dependent modifications of mitochondrial proteins comes from the disease phenotypes associated with their genetic alterations.

## 1. Introduction

It is noteworthy that cancer cells exhibit several metabolic alterations, which include increased fatty acid synthesis and glutamine metabolism, and dependence on aerobic glycolysis for energy needs [1,2], collectively referred to as the “Warburg effect” [3]. Recent evidence indicates that the Warburg effect could be a universal phenomenon in normal proliferating cells, such as mammalian early embryonic cells, stem cells, primary spermatocytes, and even in virus-infected cells, thus the study of the Warburg effect in these conditions could represent a breakthrough point, helping to elucidate the Warburg effect in cancer cells [4]. The importance of the Warburg effect in cancer has been outlined by Hanahan and Weinberg, who included the reprogramming of energy metabolism in the emerging hallmarks of cancer [5]. Among the different mitochondrial pathways involved in energy metabolism, β-oxidation of fatty acid is of particular interest because its inhibition has been suggested as a potential target to reduce tumor growth [6,7]. On the other hand, due to their role in adenosine triphosphate (ATP) production through oxidative phosphorylation (OXPHOS), mitochondria are the main intracellular producers of reactive oxygen species (ROS), which, in turn, can react with lipids and induce lipid peroxidation (LPO). Peroxidation of polyunsaturated fatty acids (PUFAs) generates an array of primary products, among which 4-hydroxynonenal (HNE) is one of the best-studied active lipid electrophiles. This aldehyde, by reacting with mitochondrial proteins, may contribute to altered mitochondrial functions not only in cancer cells, but also in other diseases, in which oxidative stress is increased, such as chronic inflammations and neurodegenerative diseases [8,9]. Moreover, both ROS and reactive aldehydes can interact with mtDNA to produce mtDNA mutations and, as a consequence, alterations in encoded proteins that are crucial for mitochondrial functions [10,11,12].

In this review, we discuss the recent findings in mitochondrial dysfunctions in cancer cells related to fatty acid composition, ROS production, LPO, and the role played by mitochondrial lipoperoxidation products in cancer and neurodegenerative disorders.

## 2. Mitochondrial Dysfunction in Cancer

### 2.1. Aerobic Glycolysis and Oxidative Phosphorylation

One well-known example of mitochondrial dysfunction in cancer cells is the cancer-associated metabolic switch towards aerobic glycolysis, known as the Warburg effect [3], which enables cancer cells to direct the utilization of glucose supplies towards the biosynthesis of macromolecules, thus supporting their rapid growth. In order to meet their energy needs, normal cells oxidize glucose via the mitochondrial tricarboxylic acid cycle (TCA), also known as Krebs cycle, to generate ~30 ATP molecules per glucose molecule. By contrast, cancer cells rely heavily on glycolysis to generate two ATPs per glucose molecule in the cytoplasm. Hence, cancer cells, such as those of gliomas, meningiomas and sarcomas, upregulate glucose transporters to increase glucose uptake into the cell, in order to meet their energy needs [13,14]. Moreover, mutations of mitochondrial enzymes (e.g., fumarate hydratase, succinate dehydrogenase and isocitrate dehydrogenase) and other key components of glycolysis and Krebs cycle are a general feature of cancer cells, leading to a reduced production of pyruvate via glycolysis and an increased flux towards biosynthetic and NADPH-producing pathways, such as serine biosynthesis and the pentose phosphate pathway [12].

It has been demonstrated that the Warburg effect is also present in normal, highly proliferating cells, in which the increase of aerobic glycolysis, associated with a reduced OXPHOS activity, could be a protective strategy against ROS [15]. Indeed, in normal cells, aerobic glycolysis attenuates OXPHOS, which is the major source of mitochondrial ROS. On the contrary, in cancer cells, despite the reduction of OXPHOS capacity [12], the level of ROS produced, mainly by the electron transfer sites, is generally increased [16] and has been associated with mtDNA mutations [4]. Overall, OXPHOS activity, even though reduced, is not suppressed in tumor cells, and mtDNA mutations seem to be functional in altering the bioenergetic and biosynthetic state of cancer cells. Also, the reduction of OXPHOS capacity is not present in all tumors: in breast cancer, upregulation of OXPHOS has been demonstrated [12], while the transcriptome analysis of pancreatic tumor-initiating cells revealed a prominent expression of genes involved in mitochondrial function and an increased consumption of oxygen. Recent studies have indeed indicated that, while glycolysis is drastically upregulated in almost all cancer cells, mitochondrial respiration continues to operate normally, at rates proportional to the oxygen supply [17]. However, OXPHOS impairment has been found in diverse cancer types and some mechanisms have been proposed: mutations of the Krebs cycle enzymes in pituitary and renal tumors [18,19], the absence of one of the principal enzymes controlling respiratory complex I in renal oncocytoma [20], and the activation of oncogenes (K-RAS), and transcription factors (HIF1-alpha) [21] in mouse transformed fibroblasts.

Since ROS content in cancer cells is generally increased, in order to maintain the steady-state concentrations of ROS, mitochondria must rely on their own antioxidant system for the scavenging and neutralization of the radicals generated by OXPHOS [22,23]. Dismutation of mitochondrial matrix superoxide (O^2−^) into H_2_O_2_ is catalyzed by Mn superoxide dismutase (MnSOD, SOD_2_), while dismutation of intermembrane space O_2_^−^, which is primarily generated by respiratory complex III [24], is catalyzed by intermembrane and cytosolic Cu/ZnSOD (also known as SOD1). Another enzyme located in the mitochondria, methylene-tetrahydrofolate dehydrogenase 2 (MTHFD2), which uses NAD^+^ as a coenzyme [25], is overexpressed in rapidly proliferating malignant tumors. It has been postulated that this enzyme constitutes the “main switch” that enables mitochondria to produce additional one-carbon units for purine synthesis in rapidly growing tumor cells, and generates the NADH necessary for the protection from ROS and macromolecular syntheses [26,27]. Interestingly, this mitochondrial enzyme is overexpressed in rapidly growing tumor tissues and not in replicating normal tissues, and the knockdown of MTHFD2 has an antiproliferative effect, suggesting that the inhibitors of this enzyme may be employed in cancer treatment [28].

### 2.2. MtDNA Mutations in Cancer Cells

Human mitochondria contain copies of a circular DNA encoding 13 polypeptide components of the OXPHOS system, 12S and 16S ribosomal RNA and 22 transfer RNAs [29]. The remaining mitochondrial proteins are encoded by the nuclear DNA. Each cell contains a number of mitochondria, and each mitochondrion contains several copies of mtDNA. Thus, the mtDNA mutations, generated by ROS attack, can be passed to daughter cells together with normal mtDNA, in a heteroplasmic state. However, it is possible that a subpopulation of cancer cells present all mtDNA copies mutated [30]. In normal mammalian cells, mtDNA has a higher mutation frequency, compared to nDNA [31,32], due to its location in the vicinity of ROS-producing OXPHOS and the lack of protective histones [33,34]. Several mtDNA mutations were reported in a wide variety of tumors. Mutations in mtDNA genes have been detected in colorectal cancer [35] and in gastric, bladder, lung, renal, prostate, and ovarian cancer [36,37,38]. However, in the majority of tumors, the high frequency and complexity of mtDNA mutations have made it difficult to understand their role in carcinogenesis [39]. Moreover, the functional interpretation of many of the mutations remains ambiguous, and few studies have demonstrated a direct relationship between mtDNA mutations and carcinogenesis. In favor of the involvement of mtDNA mutations in carcinogenesis, Petros *et al.* [40] demonstrated that mtDNA mutations play an important role in the pathogenesis of prostate cancer. These authors generated a pathogenic mtDNA T8993G mutation in the gene-encoding ATP synthase (complex V) subunit ATP6, in PC3 prostate cells, through cybrid transfer, and tested for tumor growth in nude mice. The mutant (T8993G) cybrids generated tumors that were seven times larger than the wild-type (T8993T) cybrids. Moreover, they found significantly more ROS in the mutant tumors [40]. Other authors demonstrated a direct involvement of mtDNA mutations in the induction of metastatic potential of Lewis lung carcinoma cells, fibrosarcoma cells and colon cancer cells, by using assays of metastasis in mice. The mtDNA conferring high metastatic potential contained G13997A and 13885insC mutations in the gene-encoding NADH-ubiquinone oxidoreductase (complex I) subunit ND6. These mutations produced a deficiency in respiratory complex I activity and were associated with overproduction of ROS. Interestingly, pretreatment of the highly metastatic tumor cells with ROS scavengers suppressed their metastatic potential in mice [41]. Overall, it is generally accepted that mtDNA mutations are responsible for the induction of ROS production at the expense of a loss of efficiency of the electron transfer chain. ROS, in turn, can induce mtDNA mutations and activate several signaling pathways critical for tumor growth and maintenance [42].

Besides mtDNA mutations, several studies demonstrated a variation in the number of mtDNA copies in various types of cancer [38]. However, both increases and decreases in the number of mtDNA copies have been reported to be associated with an increased risk of tumorigenesis. Thus, the role of the variations in the number of mtDNA copies in cancer is still a subject of debate.

## 3. Lipid Composition and Lipoperoxidation in Mitochondria of Cancer Cells

In mitochondria, more than in other subcellular organelles, the lipid component of membranes plays a fundamental role in regulating a wide range of mitochondrial and cellular functions, including respiration, programmed cell death, and autophagy/mitophagy. Moreover, mitochondrial lipids coordinate shifts of several proteins among subcellular compartments [43]. The importance of lipids in controlling mitochondrial functions is further confirmed by several observations: (1) lipid types, in particular phospholipid classes, differ in the two mitochondrial membranes (outer mitochondrial membrane, OMM, and inner mitochondrial membrane, IMM), indicating a specific correlation between lipid composition and the functions that they carry out; (2) mitochondria have the capacity to synthesize some phospholipid classes; (3) mitochondria from different cell types and different species share the above characteristics.

### 3.1. Lipid Composition and Mitochondrial Functions in Normal and Cancer Cells

In both the outer (OMM) and the inner (IMM) mitochondrial membrane, the main phospholipids are phosphatidylcholine (PC), phosphatidylethanolamine (PE), phosphatidylinositol (PI), phosphatidylserine (PS), and phosphatidic acid (PA). Besides these major phospholipids classes, which are also present in other cellular membranes, mitochondrial membranes exclusively contain cardiolipin (CL) and phosphatidylglycerol (PG). Unlike in other cell membranes, the percentages of sphingolipids and cholesterol in mitochondrial membranes are rather low [44,45]. The differences in percentage content of phospholipids classes in OMM and in IMM are reported in Table 1 [46].

OMM and IMM also differ from other cell membranes with regard to the protein/lipid ratio. In general, in mitochondria this ratio is lower than in other subcellular organelles, and IMM shows a higher protein level and a lower lipid content than OMM [45,46]. In spite of the low lipid content in IMM, it is known that mainly CL and PE are crucial for the activity of several mitochondrial proteins, including adenine nucleotide translocator and ATP synthase, and functions, such as oxidative phosphorylation, mitophagy, and apoptosis [47,48].

As already mentioned, mitochondria have the capacity to synthesize some phospholipid classes, in particular CL, PE, PG, and PA. The synthesis of CL takes place in the inner (matrix) side of IMM and is regulated by several factors. PE is formed by decarboxylation of PS in the IMM, and this process produces almost all the required PE. Moreover, the PE produced can be exported out of the mitochondria. The import/export of phospholipids into and out of the mitochondria can occur in different ways: (1) spontaneous transport; (2) via contact sites in the membranes; (3) via the activity of phospholipids scramblases (flippases) responsible for the bidirectional phospholipid transport; (4) via vesicular transport [49,50,51,52]. The different phospholipid classes are not evenly allocated in mitochondrial membranes, but they show an asymmetric distribution. For example, in IMM the percent localization of PC and PE is similar in the side facing the intermembrane space and the matrix side, whereas CL and PI are mainly (about 80%) located in the side facing the matrix [46,53].

Both biochemical and functional properties of different phospholipid classes are strongly influenced by their fatty acid component. It is well known that in most mitochondria, linoleic acid (LA) is the main PUFA in CL (about 80%), and that a decreased percentage content of LA negatively affects COX activity [54,55,56]. Differently, in brain mitochondria, the acyl moiety of CL mainly consists of long chain PUFAs, including docosahexaenoic acid (DHA, C22:6) and arachidonic acid (AA, C20:4) [57]. Saturated palmitic acid (C16:0) is the most abundant fatty acid in testicular CL, and probably protects spermatozoa from LPO [58,59]. The fatty acid composition of mitochondrial membrane phospholipids is known to be influenced by two main factors: the intra-mitochondrial fatty acid synthesis and the pattern of fatty acids present in the diet [60]. Eukaryotic cells possess a highly conserved mitochondrial pathway for fatty acid synthesis, which is completely independent of the one that is working in the cytoplasm. The mitochondrial machinery synthesizing fatty acids consists of mono-functional polypeptides not yet well characterized in human beings [61,62]. Nevertheless, it has been reported that these enzymes show a wide substrate specificity, and act on short-chain substrates (C2 up to C16) [62].

With regard to the importance of dietary fatty acids, several studies have evidenced that changes in the fatty acid content of food significantly modify mitochondrial phospholipids, and in particular CL properties, the effects being also related to the different types of cells. Conversely, a recent lipidomic-based analysis concluded that fatty acid content in CL does not directly reflect the dietary fatty acid content, but is rather a result of a specific selections [63]. The analysis of the changes occurring in mitochondrial membrane phospholipids in aging evidenced an increased content of DHA-containing phospholipids, mainly in PC and in PE [64]. Overall, the most important role in modulating fatty acid composition and functions in mitochondria seems to be played by dietary PUFAs, and in particular by *n*-3 ones, even though the effects are once again related to phospholipid classes and cell types. In general, PC seems to be more affected by the variation of dietary monounsaturated fatty acids (MUFAs), whereas PE is more responsive than PC to the dietary content of n-6 and n-3 PUFAs [65].

Concerning the importance of the differences in fatty acid composition of mitochondria from various tissues, it has been reported that the presence of four linoleic acyl chains in CL is essential for the optimal function of heart mitochondria; moreover, it has been recently observed that *n*-3 PUFA supplementation, even though increasing the production of ROS, did not induce oxidative damage and favored mitochondrial membrane reorganization in human skeletal muscle [66]. In agreement with these studies, a high intake of DHA- and EPA-enriched fish oil prevented the age-associated increase of PC [67].

In the light of the importance of phospholipid classes and of fatty acid content in regulating mitochondrial and overall cellular functions, the changes in lipid composition in carcinogenesis have been investigated, mainly in animal models and in cultured cancer cells. The reported modifications in the mitochondrial lipid profile concerned both neutral and polar lipids. In mitochondria from hepatocarcinoma, the following changes have been observed: (1) an increase in cholesterol/phospholipid ratio; (2) a decreased content of polyenoic acyl chains in phospholipids; (3) a diminished ability to undergo volumetric and conformational changes; (4) an increased lability of the phosphorylating machinery.

The modifications of the fatty acid composition of phospholipid classes observed by Canuto *et al.* [68] in mitochondrial membranes during diethylnitrosamine carcinogenesis in rat liver are reported in Table 2.

The reported results evidenced that changes in the MUFA/PUFA ratio are an early event in hepatocarcinogenesis, already detectable in preneoplastic lesions (nodules), and that a MUFA increase and PUFA decrease are present also in highly deviated AH-130 ascites hepatoma. Conversely, no significant change in the fatty acid profile has been observed by other authors in the mitochondria isolated from rat liver nodules induced by a 2-acetylaminofluorene-containing diet [69]. It is possible that this discrepancy is due to the different times at which the nodules were analyzed; in fact, in the first study, fatty acid analysis was carried out between 7 and 10 months after exposure to the carcinogenic agent, and after 25 weeks in the second study.

The increased MUFA and decreased PUFA content, reported by Canuto *et al.* [68], have been observed also in some types of Morris hepatomas showing different growth rates and malignancy degrees [70]. In particular, a decreased content of AA has been observed in total phospholipids extracted from normal rat hepatocytes and rat hepatoma cell lines 7777 and JM2 [71].

It was speculated that the previously reported modifications in phospholipid classes and fatty acid content in mitochondria from cancer cells might be responsible for several characteristics of cancer cells, including their different susceptibility to mitochondrial-driven apoptosis. In this regard, it has been evidenced that the incorporation of PUFAs in cardiolipin is able to induce apoptosis via transition pore opening, mitochondrial potential decrease and caspase-9 and caspase-3 activation [72].

### 3.2. Lipid Catabolism and Reactive Oxygen Species (ROS) Production in Cancer Cells

**Figure 1:**
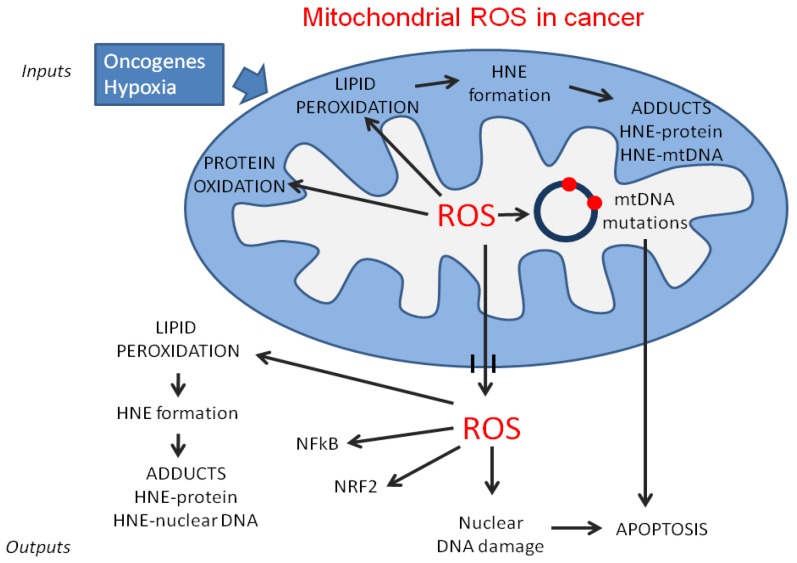
Effects of mitochondrial ROS in cancer cells.

Lipid catabolism in cancer cells is generally increased, as demonstrated in human hepatocarcinoma HepG2 cells, where lipid catabolism may replenish the mitochondrial acetyl-CoA pool required for OXPHOS [73]. Moreover, high carnitine-acyl-transferase 1 (CPT1) mRNA contents have also been found in ovary, colon, esophageal, prostate and colorectal carcinomas, as well as in Zadjela hepatoma and K-RAS transformed fibroblasts [74,75,76]. These results show that β-oxidation of mitochondrial free fatty acids is certainly functional in cancer cells, driving the OXPHOS-dependent ATP supply for cell proliferation. Nevertheless, some results from other groups did not seem to be in agreement with these observations. For example, during tumor cell proliferation, growth-factor signaling suppressed β-oxidation of fatty acids, minimizing futile cycling and maximizing lipid synthesis [77]. Thus, it has been suggested that fatty acid oxidation in cancer cells may be context-dependent or cancer cell-type specific [78]. The changes in fatty acid β-oxidation, together with other variations affecting the integration between metabolic pathways, which in part can involve mitochondria, such as the levels of amino acid intermediates and the activity of the pentose phosphate pathway, likely entail redox state perturbations. Moreover, hypoxia, caused by inadequate oxygen supply from the local vasculature, results in increased generation of ROS and cell death [79]. As a consequence, the extent of the alterations induced by ROS and by their peroxidative products on whole cells and mitochondria is increased in cancer cells. One of the targets of ROS is mitochondrial mtDNA, which encodes several proteins important for the function of the mitochondrial electron transport chain and for ATP synthesis by OXPHOS [29]. As mentioned before, mtDNA is highly susceptible to attack by ROS, owing to its close proximity to the electron transport chain, the major site of free radical production, and the lack of protective histones. Therefore, mtDNA oxidative damage could lead to the loss of mitochondrial electron transport, membrane potential and ATP generation [80]. Moreover, the peroxidation of PUFAs in mitochondrial membranes induced by ROS leads to the production of reactive aldehydes which, in turn, can contribute to the inhibition of enzyme activities, as demonstrated by Raza *et al.* [81]. It has been postulated that high HNE concentrations can also alter mitochondrial functions through the formation of adducts with mtDNA, similar to those observed in nuclear DNA [82]. Oxidative damage, directly or indirectly induced by ROS, is probably the major source of mitochondrial genomic instability leading to respiratory alterations, and is responsible, among other consequences, for the mitochondrial dysfunctions observed in cancers, particularly in the most aggressive and rapidly growing ones (Figure 1) [80,81,82,83].

In cancer cell mitochondria the level of ROS is generally increased, due to multiple inputs that regulate the generation of ROS (e.g., hypoxia and oncogenes). The mitochondrial ROS generate mtDNA mutations, leading to mitochondrial dysfunctions and apoptosis, protein oxidation and lipid peroxidation. Lipid peroxidation leads to the production of aldehydes, in particular HNE which, in turn, can bind and inactivate mitochondrial proteins. ROS released from mitochondria can induce lipid peroxidation of cellular membranes, nuclear DNA damage, which contribute to the apoptosis induction, and transcription factor activation.

### 3.3. Lipid Peroxidation and Its Products

Some ROS can oxidize virtually any biological macromolecule, but PUFAs represent the main target of intracellular oxidizing agents. Many of the products of lipid oxidation are chemically reactive and capable of inducing alterations in cell signaling and survival, through their ability to modify various cellular targets [84]. Among them, the reactive HNE is the most studied because of its highest biological activity. HNE is derived from the decomposition of *n*-6 PUFAs. Phospholipids containing LA (18:2, *n*-6) and AA (20:4, *n*-6) in extra mitochondrial membranes are considered the major source of HNE production [85]. However, HNE can be produced in mitochondria too. Indeed, it has been demonstrated that the IMM is rich in CL, which contributes greatly to its unsaturated nature. The acyl chain composition of CL in humans and rodents includes primarily LA, the relative abundance of this fatty acid in mouse CL ranging from 60% in skeletal muscle to more than 80% in the heart and the liver [86]. Several experimental results demonstrated that the oxidation of mitochondrial CL also leads to the formation of a significant amount of HNE, MDA and other oxidation products [87,88]. It has been shown that the reaction of HNE with hepatic mitochondrial membranes results in altered lipid-lipid and protein-lipid interactions. This occurs through a variety of mechanisms, including cross-linking of lipid tails, which limits phospholipid mobility in the lipid bilayer [89]. Overall, the studies on the effects of LPO on membrane physiology demonstrate a marked reduction in membrane fluidity [90,91] and an increase in membrane permeability [92,93]. Alterations of mitochondrial membranes, mainly due to the increased mitochondrial LPO, have been detected after pro-oxidant cisplatin treatment in kidney and Dalton’s lymphoma cells, which showed more roundish mitochondria with thickened membranes, numerical reductions and irregularities in the shape of cristae and intramitochondrial vacuoles [94]. However, no data have been provided thus far on the perturbations of mitochondrial membrane functions possibly associated with the above-described modifications.

### 3.4. 4-Hydroxynonenal (HNE)-Protein Adducts in Cancer Cell Mitochondria

HNE can easily react with several cellular proteins and affect their function [95]. In particular, it has been demonstrated that mitochondrial proteins can represent a suitable target for the production of HNE adducts [88]. By using a proteomic approach, involving two-dimensional electrophoresis, followed by mass spectrometry and investigation of protein databases, Zhao *et al.* [96] identified several HNE-modified mitochondrial proteins in cardiac mitochondria from doxorubicin-treated mice. The majority of the identified proteins were related to mitochondrial energy metabolism, such as certain enzymes of the citric acid cycle and the electron transport chain, whose modifications negatively affected their enzymatic activities and the respiratory function of cardiac mitochondria. Treatment with Mn (III) meso tetrakis (*N*-*n*-butoxyethylpyridinium-2-yl) porphyrin, a SOD mimic, averted the doxorubicin-induced mitochondrial dysfunctions, as well as the formation of HNE-protein adducts. Functional inactivation is a common consequence of the covalent modification of enzymes by HNE. Other results by Doorn *et al.* showed that HNE inhibited mitochondrial aldehyde dehydrogenase 2 (ALDH2), as a consequence of the formation of a HNE-cysteine adduct at the ALDH2 active site [97]. Studies from Asian countries evidenced a significant association between ALDH2 enzyme deficiency and esophageal cancer risk [98]. It might be speculated that HNE might participate in carcinogenesis, also due to its inhibitory effect on ALDH2 activity. The interaction of HNE with cellular proteins has been evoked in support of both its carcinogenic and anti-carcinogenic roles, depending on the particular HNE-modified protein and the biological consequences of its modification [9,84,99]. However, the ability of HNE to alter mitochondrial function is not always a consequence of HNE-mitochondrial protein adduct formation, but it might also depend on the reduction of the cellular content of detoxifying molecules, such as GSH, resulting in the maintenance of high intracellular levels of ROS [100]. This hypothesis has been confirmed by Raza *et al.* [81], who found that HNE treatment of PC12 pheochromocytoma cells caused a reduction in the GSH pool and a marked inhibition in the activities of mitochondrial enzymes cytochrome c oxidase and aconitase. Until now, comprehensive studies of HNE-protein adducts and of HNE effects on mitochondrial functions in diverse cancer types have not been presented. The presence of nuclear and mitochondrial HNE-protein adducts in both normal proximal renal tubule and in three types of renal carcinoma has been reported, but no characterization of the proteins involved and of the functional consequences of adduct formation has been provided [101]. Mitochondrial HNE-protein adducts have been found in a multistage skin carcinogenesis model during papilloma formation, suggesting that the presence of these adducts might be involved in cutaneous carcinogenesis [102].

Specific HNE protein adducts have been identified by Li *et al.*, who showed that HNE inhibited, by thiol-specific modification, the deacetylase activity of Sirtuin-3 (SIRT3), a major mitochondrial NAD^+^-dependent deacetylase, leading to increased angiogenesis and invasion of breast cancer cells [103]. Zhang *et al.* demonstrated that the thioredoxin reductase (TrxR), the only known enzyme catalyzing Trx2 reduction in mitochondria, was sensitive to inactivation by HNE, and that this effect might account for the induction of apoptosis by HNE in HeLa cancer cells. [104]. Other investigators showed that HNE was able to trigger apoptosis of human colorectal carcinoma (RKO) cells and RAW 264.7 cells, through a mitochondrion-dependent pathway [105,106].

An overview of the effects of HNE on mitochondrial proteins and functions in different types of cancer cells is provided in Table 3.

Moreover, although increased oxidative stress has been demonstrated in the majority of cancer types [99], the concentration of LPO products, such as HNE, was not increased in every one of them, possibly varying in relation with the composition of cell membranes and metabolizing enzymes. Indeed, both decreased and increased HNE concentrations, compared with normal tissues, have been reported in cancer tissues, depending on the tumor type or the presence of inflammation [69,72,84]. In conclusion, even though increased ROS production and ROS-induced mtDNA mutations have been found to be related to carcinogenesis, the presence of covalent modifications of mtDNA with HNE and other lipid electrophiles and the direct involvement of HNE adducts with mitochondrial proteins in the pathogenesis of cancer remain aspects that still require further investigation.

## 4. Oxidative Stress and Mitochondrial Dysfunction in Neurodegenerative Disease

Oxidative stress is implicated in the pathogenesis of most neurodegenerative diseases, including Alzheimer’s disease (AD), tauopathies and alpha-synucleinopathies, *i.e.*, Parkinson’s and related diseases (PD) and Huntington’s disease (HD). Oxidative damage occurs in early stages of AD [107,108,109,110]. Oxidative alterations of proteins by ROS have been implicated in the progression of neurodegenerative disorders. In fact, high levels of markers of LPO have been found in brain tissues and body fluids in the above-mentioned diseases [111,112,113,114]. Novel 2,4-diphenylhydrazine (DPNH)-reactive carbonyl groups in proteins, susceptible to spectrometric or antibody-mediated detection, might be either produced by the direct metal-catalyzed oxidation of aminoacyl side chains or introduced by stable adduct formation via the reaction of lysyl residues with reducing sugars and third-party reactive carbonyl species (RCS). As some of these, like glyoxal and methylglyoxal, might derive from free-radical attack to derivatives of both carbohydrate and lipid metabolism [115], their adducts with lysyl residues of proteins are referred to as mixed advanced glycoxidation end products (AGEs)/advanced lipoxidation end products (ALEs) [116]. Typical LPO products, formed upon the free-radical attack to PUFAs, include acrolein, MDA and HNE. They exert toxic effects upon cells, by altering several of their functions, mostly by forming covalent adducts with cell proteins [117]. The immunodetection of protein-bound *N*-(malondialdehyde)-lysine (MDAL) and HNE adducts with cysteinyl, histidyl and lysyl residues in proteins has been largely exploited as a measure of lipid peroxidation in neurodegenerative diseases [116,118,119]. Inventories of modified cellular proteins were compiled by several authors, who investigated the consequences of increased oxidation, glycoxidation or lipoxidation in the course of oxidative stress, by the means of redox proteomic approaches, combining bidimensional gel electrophoresis, Western blotting with oxidative markers and mass spectrometry [107,109,116,119,120,121]. Vulnerable proteins could be assigned to a few distinct functional groups, with crucial roles in membrane transport, energy metabolism, mitochondrial function, antioxidant defenses, cellular stress responses, cell signaling, signal transduction, cytoskeletal organization, protein synthesis and neurotransmission (reviewed in [108,120,122,123]). It appears from these studies that in neurodegenerative diseases the generation of adducts of LPO products with several cell proteins is associated with losses of specific functions, which, in turn, might cause the progressive endangerment of vital processes, ultimately leading to neuronal death [93]. Moreover, in AD, by reacting directly also with amyloid beta peptide (Aβ), HNE exacerbated the aggregation and toxicity of Aβ oligomers, which, in turn, enhanced oxidative stress and LPO, thus fueling a vicious circle of toxic Aβ oligomer formation [108,120,124].

The involvement of mitochondria in the pathogenesis of neurodegenerative disorders has been suggested in consideration both of the mtDNA mutations that can be caused by ROS [125,126], and of the frequent participation of several mitochondrial proteins in the formation of covalent adducts of LPO products. We focus here on the structural and functional consequences of these modifications.

## 5. Role of the Adducts of Aldehydes Derived from Lipid Peroxidation with Mitochondrial Proteins in Neurodegeneration

The detection of protein adducts with LPO products by redox proteomic approaches was often only circumstantially associated with a disease. Functional studies of target proteins, especially with regard to adduct formation in the disease context, have not been always provided. In an attempt to investigate further the possible impact of the oxidative modifications of mitochondrial proteins on the neuronal dysfunctions observed in neurodegenerative diseases, we provide here, in addition to an account of the adducts of LPO products with mitochondrial proteins so far identified and the related functional studies, whenever present in the literature (Table 4), also a description of the mutations of the respective genes and the associated phenotypes, retrieved in the Online Mendelian Inheritance in Man (OMIM) database [127]. 

The concept at the base of this approach is that genetic mutations and oxidative modifications of proteins may represent different ways of altering the integrity of cell proteins, to a point where disease manifestations occur. The genetic and acquired modifications of Cu, Zn-superoxide dismutase (SOD1), a key antioxidant enzyme whose mutations have been linked to the autosomal dominant familial form of amyotrophic lateral sclerosis (ALS), may represent a reference model for this hypothesis. The SOD1 pI 6.0 isoform was found to be oxidatively modified by carbonylation in human brain post-mortem samples from AD and PD patients. Moreover, Cys146, a residue of SOD1 that is mutated in familial ALS, was oxidized to cysteic acid in the same brain samples. In AD brains, SOD1 formed proteinaceous aggregates associated with amyloid senile plaques and neurofibrillary tangles. These data implicate oxidative damage to SOD1 in the pathogenesis of sporadic AD and PD [133].

### 5.1. HNE-Modified Mitochondrial Proteins

#### 5.1.1. Mitochondrial Aconitate Hydratase (Aconitase 2, ACO2)

Mitochondrial aconitate hydratase (aconitase 2, ACO2) catalyzes step 2 in the Krebs cycle, *i.e.*, the reversible isomerization of citrate to isocitrate, via the dehydration of citrate to *cis*-aconitate, followed by the hydration of *cis*-aconitate to isocitrate. There is an extensive documentation on ACO2 as a target of oxidative stress, due to the high sensitivity of its Fe-S cluster (reviewed in [134]). Chen *et al.* showed that yeast mitochondrial aconitase is essential for mitochondrial DNA maintenance, independent of its catalytic activity [135]. HNE adducts with ACO2 were detected in the hippocampus of patients with late-stage AD (LAD), in 185% excess over control brain samples; investigation of the functional consequences of the modification revealed that the enzymatic activity of ACO2 was reduced by 50%, supporting the view that oxidative modification determined functional impairment [121].

In this context it is of interest that the mutations of the *ACO2* gene are associated with infantile cerebellar-retinal degeneration (ICRD, MIM 614559), a severe autosomal recessive, neurodegenerative disorder, characterized by onset between ages two and six months of truncal hypotonia, athetosis, seizures and ophthalmologic abnormalities, such as optic atrophy and retinal degeneration. Affected individuals show profound psychomotor retardation, with only some achieving rolling, sitting, or recognition of family. Brain MRI shows progressive cerebral and cerebellar degeneration. Allelic mutations of the same gene are also associated with optic atrophy 8 (OPA 8, MIM 616289), a progressive visual loss in association with optic atrophy, reflecting a deficiency in the number of nerve fibers which arise in the retina and converge to form the optic disk, optic nerve, optic chiasm and optic tracts.

#### 5.1.2. Mitochondrial Adenosine Triphosphate (ATP) Synthase (Respiratory Complex V), Alpha Subunit 1 (ATP5A1)

ATP synthase (Respiratory Complex V, EC 3.6.3.14) is a multimeric complex located in the IMM, consisting of two fractions (F_1_ and F_O_) and at least 16 polypeptides, two of which (MT-ATP6, MT-ATP8) are encoded by mitochondrial genes and the remainder by nuclear genes. The F_O_ (oligomycin-binding) fraction is a proton pore embedded within the inner mitochondrial membrane, while the F_1_ fraction projects into the mitochondrial matrix. ATP synthase catalyzes the synthesis of ATP from ADP and inorganic phosphate by coupling it with a flow of protons from the mitochondrial intermembrane space to the matrix.

Mitochondrial dysfunction, with reduced glucose utilization and decreased ATP synthase activity and energy production, is an early occurrence in the pathogenesis of AD [136,137]. Significant increases in the levels of HNE-modified ATP synthase alpha subunit, accompanied by a 30% reduction in ATP synthase activity (at variance with respiratory complex I, whose activity was unaltered), were observed in post-mortem homogenates of entorhinal cortex in early, clinically silent cases of AD in Braak stages I/II, in comparison with control samples [128]. ATP synthase alpha chain was also one of several proteins with markedly increased levels of HNE adducts in the hippocampus (660% over control levels) and in the inferior parietal lobule (IPL) (450% over control levels) of patients with amnestic mild cognitive impairment (MCI), a condition of increased risk for AD. ATP synthase alpha subunit was the only HNE-modified protein identified in both anatomical locations. ATP synthase activity was reduced by 35% in the hippocampus and by 30% in the IPL of MCI brains, compared with controls [109]. The relative increase of HNE-modified ATP synthase alpha chain in the IPL of patients with early AD (EAD) was marginal, with a 20% activity reduction [110], while in late-stage AD (LAD) the relative increase of HNE-modified ATP synthase alpha chain in the IPL was 250% over control samples, while ATP synthase activity was decreased by 48% and the protein level was also decreased in comparison with controls, suggesting that the oxidative impairment of ATP synthase activity might be accompanied by downregulation of the expression or destabilization of the protein product [121].

Taken together, these data favor the view that the modification of cell proteins with LPO products is an early event in the progression of AD and is associated with impairments of enzymatic activity and cell functions. Furthermore, it was suggested that oxidation of ATP synthase alpha subunit, besides compromising ATP synthesis, might also enhance ROS production, resulting in a vicious cycle of mitochondrial dysfunction and neuronal damage [128,138].

Mutations of the *ATP5A1* gene, encoding the alpha subunit of the ATP synthase F_1_ fraction, are associated with two phenotypes of human disease: mitochondrial complex V (ATP synthase) deficiency, nuclear type 4 (MC5DN4, MIM 615228), which is an autosomal recessive, fatal form of infantile encephalopathy, and combined oxidative phosphorylation deficiency 22 (COXPD22, MIM 616045), also an autosomal recessive disorder, resulting in early death.

Several other types of complex V ATP synthase deficiency have been described, in association with the mutations of nuclear and mitochondrial genes encoding other complex V subunits. Among them, most interesting is ATP synthase deficiency, mitochondrial type 1 (MC5DM1), associated with mutations of the *MTATP6* gene and with several disease phenotypes:

(a) Leigh syndrome (LS, Necrotizing encephalopathy, infantile subacute, SNE, MIM 256000). This is an early-onset, progressive neurodegenerative disorder, with bilateral foci of demyelination, necrosis, gliosis, spongiosis or capillary proliferation in one or more areas of the central nervous system (brainstem, thalamus, basal ganglia, cerebellum and spinal cord). Patients exhibit psychomotor retardation, epilepsy, cerebellar and motor disturbances, extraocular muscle incoordination, neurogenic breathing disorders, neural deafness, retinitis pigmentosa, polyneuropathy, lactic acidosis, and cardiomyopathy. LS is genetically heterogeneous, being associated with the defects of several mitochondrial- and nuclear-encoded subunits and assembly factors of respiratory complexes IV, I (most often), but also V, III, mitochondrial tRNAs and other nuclear-encoded mitochondrial proteins, such as pyruvate dehydrogenase E-1α subunit.
(b) LHON (Leber Hereditary Optic Neuropathy, MIM 535000). LHON presents in midlife as acute or subacute central vision loss, with peripapillary microangiopathy and telangiectasia, to central scotoma and blindness, inherited with a maternal pattern of transmission. Disturbances of cardiac conduction and dystonia may coexist. The disease has been associated with many allelic missense mutations in the mtDNA that can act autonomously or in association with each other to cause the disease. LHON is also genetically heterogeneous, possibly reflecting mutations of most mitochondrially encoded subunits of respiratory complexes I, III, IV and V.
(c) NARP (Neurogenic muscle weakness, Ataxia, and Retinitis Pigmentosa, MIM 551500). This syndrome entails a combination of developmental and psychomotor retardation, retinitis pigmentosa, dementia, seizures, clonic spasms, ataxia, proximal neurogenic muscle weakness, sensory neuropathy, hearing loss and optic atrophy, with a maternal pattern of transmission, with no histochemical evidence of myopathy. Mattiazzi *et al.* [139] showed that the T8993G mutation of the *ATP6* gene inhibited oxidative phosphorylation and enhanced free radical production. Antioxidants restored respiration and partially rescued ATP synthesis in cells harboring the mutation, suggesting that free radicals may play an important pathogenetic role.

#### 5.1.3. Mitochondrial Translation Elongation Factor Tu (EF-Tu, TUFM), Malate Dehydrogenase 2 (MDH2), and Mn Superoxide Dismutase (SOD2)

Translation elongation factor Tu (EF-Tu, TUFM) promotes the GTP-dependent binding of aminoacyl-tRNA to the A-site of ribosomes during protein biosynthesis. Marked increases (>300%) of the amount of HNE-modified EF-Tu was detected in post-mortem homogenates of IPL from patients with amnestic MCI, in comparison with control brains, but no activity measurements were performed [109]. Mutations of the *TUFM* gene are associated with combined oxidative phosphorylation deficiency 4 (COXPD4, MIM 610678), a disease resulting in severely decreased mitochondrial protein synthesis and combined deficiency of mtDNA-encoded respiratory chain complex subunits, with neonatal onset of lactic acidosis and rapidly progressive encephalopathy.

Mitochondrial malate dehydrogenase 2 (MDH2) catalyzes the last step in the Krebs cycle, *i.e.*, the reversible oxidation of malate to oxaloacetate. This enzyme plays a key role also in gluconeogenesis, by reducing mitochondrial oxaloacetate to malate, which is transported into the cytosol, to be oxidized back to oxaloacetate. Marked increases (>500%) in the levels of HNE-modified MDH2 in post-mortem IPL homogenates from patients with EAD, in comparison with control samples, were reported in association with a 160% increase of enzymatic activity [110]. There are no known human disease phenotypes associated with the mutations of the *MDH2* gene.

Mn superoxide dismutase (SOD2) detoxifies cells by destroying excess superoxide anion radicals. Marginal increases in the levels of HNE-modified SOD2 were detected in the IPL of patients with EAD, in comparison with controls. These were accompanied by a 45% activity reduction [109]. In contrast, the amount of HNE-modified SOD2 in the IPL of patients with LAD was 170% over control values; the protein levels of this enzyme where also increased, while the enzymatic activity did not show significant changes [121]. Variations of the *SOD2* gene affect the individual susceptibility to microvascular complications of diabetes 6 (MVCD6, MIM 612634), a combination of pathological conditions characterized by increased vascular permeability, tissue ischemia and angiogenesis, developing in various tissues and organs as a consequence of diabetes mellitus, and including diabetic retinopathy, nephropathy, and neuropathy.

### 5.2. Malondialdehyde-Modified Mitochondrial Proteins

#### Ubiquinol-Cytochrome c Reductase (Respiratory Complex III, Cytochrome B-C1) Core Protein 1 (UQCRC1), ATP Synthase (Respiratory Complex V) Beta Subunit (ATP5B), and 60-kDa Heat Shock Protein (HSPD1, HSP60, GroEL), and Mitochondrial Glutamate Dehydrogenase 1 (GDH1)

These four enzymes will be discussed together, as all of their oxidative modifications were reported in the same detailed study of the products generated by diverse oxidative pathways (direct metal-catalyzed oxidation, reaction with mixed AGEs/ALEs and with LPO products) in post-mortem brain samples from eight AD patients and five age-matched controls. In particular, *N*-(MDA)-lysine adducts with UQCRC1 and ATP5B were found in AD brains at concentrations largely exceeding (>400%) those found in control brains, while only slight increases of the *N*-(MDA)-lysine adducts with HSP60 and no increases of the corresponding adducts with GDH1 were observed. No functional characterization of the oxidized enzymes was provided [116].

Ubiquinol:ferrocytochrome c oxidoreductase (Respiratory Complex III, EC 1.10.2.2), located within the IMM, catalyzes the second step in the electron transport chain, *i.e.*, the transfer of electrons from ubiquinol (reduced coenzyme Q10) to cytochrome c, coupled with proton translocation from the mitochondrial matrix to the intermembrane space. Complex III was resolved into 11 polypeptides by SDS-PAGE. Mutations of the *UQCRC1* gene have not been reported. Instead, several mutations in nuclear genes encoding other complex III subunits, assembly factors, and chaperones have been described. The most severe neurodegenerative disorders, with encephalopathy and psychomotor retardation or regression, are nuclear types 1, 2, 4, 7, 8 and 9 of mitochondrial complex III deficiency, respectively associated with mutations of the *BCS1L*, *TTC19*, *UQCRQ*, *UQCC2*, UQCC3 and *LYRM7* genes, respectively encoding mitochondrial chaperone BCS1, mitochondrial tetratricopeptide repeat protein 19 (both required for complex III assembly), cytochrome b-c1 subunit 8, ubiquinol-cytochrome-c reductase assembly factors 2 and 3, and LYRM7 (an assembly factor for Rieske Fe-S protein). Mitochondrial type complex III deficiency can also be caused by mutations of the *MTCYB* gene, encoding cytochrome b, the only mitochondrial-encoded subunit of complex III. Disease alleles are associated with phenotypes of LHON (Leber’s hereditary optic neuropathy, MIM 535000), or of exercise intolerance, isolated or in association with encephalomyopathy, cardiomyopathy, or multisystem disorder (growth and mental retardation, epilepsy, deafness, retinitis pigmentosa).

ATP synthase beta subunit is part of the F_1_ fraction of the ATP synthase complex (Respiratory Complex V, EC 3.6.3.14). The contribution of the oxidative damage of mitochondrial ATP synthase (Respiratory Complex V) to neurodegeneration has been discussed above, in relation with the adducts of HNE with the ATP synthase alpha subunit detected in AD. Mutations of the *ATP5B* gene have not been described in association with the inherited defects of respiratory complex V.

HSP60 functions as a chaperone in mitochondrial protein import and macromolecular assembly. It may also prevent misfolding and promote the refolding and proper assembly of unfolded polypeptides generated under stress conditions in the mitochondrial matrix. Mutations of the *HSPD1* gene are associated with spastic paraplegia 13, AD (SPG13, MIM 605280), a neurodegenerative disorder characterized by a slow, progressive weakness and spasticity of the lower limbs, whose rate of progression and clinical severity can vary [140]. In some forms of the disorder, urinary incontinence may appear, or the weakness and stiffness may spread to other parts of the body. Allelic mutations of the *HSPD1* gene cause leukodystrophy, hypomyelinating, 4 (HLD4, MIM 612233), a severe autosomal recessive, hypomyelinating leukodystrophy, characterized by infantile-onset rotary nystagmus, progressive spastic paraplegia, neurologic regression, motor impairment, profound mental retardation, with death occurring within the first two decades.

Mitochondrial glutamate dehydrogenase 1 (GDH1) plays a key role in glutamine anaplerosis, by converting L-glutamate into alpha-ketoglutarate, an important intermediate in the tricarboxylic acid cycle. Mutations of the *GDH1* gene are associated with familial hyperinsulinemic hypoglycemia 6 (HHF6, congenital hyperinsulinism, MIM 606762), the most common cause of infantile persistent hypoglycemia, due to defective feedback regulation of insulin secretion by glucose.

### 5.3. Mitochondrial Proteins with Increased Content of DPNH-Reactive Groups

#### 5.3.1. Mitochondrial Aconitate Hydratase (Aconitase 2, ACO2)

Increased contents of protein carbonyls were detected by Western blot after derivatization with DNPH in aconitate hydratase (aconitase 2, ACO2), as well as in glial fibrillary acidic protein (GFAP), γ-enolase and ubiquitous mitochondrial creatine kinase (CKMT1A), in a proteomic and enzymatic analysis of human brain post-mortem samples from the striatum and cortex of eight patients with Huntington’s disease (HD) and as many matched controls. Notably, ACO2 showed a selective, se in protein level and a 35% decrease of enzymatic activity in the striatum, but not in the cortex of HD patients, suggesting that the loss of activity could be a consequence both of reduced protein levels and of inactivation by oxidative modification [129]. It had been reported previously that ACO2 activity was decreased to 8% in HD caudate, 27% in putamen and 52% in cerebral cortex (being normal in HD cerebellum and fibroblasts), and was more sensitive to inhibition by nitric oxide (NO), in comparison with respiratory complexes II and III, already known to be vulnerable to it. It was hypothesized that a self-amplifying cycle of ROS generation and aconitase inhibition, triggered by NO and resulting in severe ATP depletion, might be critical for neuronal cell death and HD pathogenesis [141]. These results were in keeping with those reported later in relation to AD [121] and described in 5.1.1, where the disease phenotypes associated with the mutations of the *ACO2* gene have also been described.

#### 5.3.2. Mitochondrial Citrate Synthase (CS), Ubiquitous Creatine Kinase (CKMT1A), Ubiquinol-Cytochrome c Reductase (Respiratory Complex III, Cytochrome B-C1) Core Protein 2 (UQCRC2), and ATP Synthase (Respiratory Complex V) Alpha Subunit 1 (ATP5A1)

These four enzymes will also be discussed together, as their oxidative modifications in HD were reported in a single study. In a refinement of the above-cited study [129] of protein carbonylation in the striatum of HD patients, Sorolla *et al.* identified 13 carbonylated, low-abundance proteins, seven of which were involved in energy production, including three glycolytic and four mitochondrial enzymes, namely, citrate synthase (CS), ubiquitous creatine kinase (CKMT1A), ubiquinol-cytochrome c reductase (complex III) core protein 2 (UQCRC2) and ATP synthase alpha subunit 1 (ATP5A1) [130]. Mitochondrial CS is involved in the first step of the Krebs cycle, *i.e.*, the synthesis of isocitrate from oxaloacetate, which precedes the reversible isomerization of isocitrate to citrate catalyzed by ACO2. CKMT1A catalyzes the reversible transfer of phosphate between ATP and various phosphogens, like creatine phosphate); its isoenzymes play a central role in tissues with large, variable energy demands, such as skeletal muscle, heart and brain. In order to determine whether oxidation might lead to decreased function, the activities of CS, CKMT1A and ATP synthase were measured in mitochondrial-enriched striatal homogenates from HD and control subjects. All three mitochondrial enzymes exhibited significantly decreased activities in HD samples, compared to controls, in agreement with the mitochondrial dysfunctions observed in HD patients [142].

There are no known disease phenotypes associated with the mutations of mitochondrial CS and CKMT1A. The mutations of the genes encoding various subunits of ubiquinol-cytochrome c reductase (complex III) have been described in point 5.2.2. A homozygous mutation of the *UQCRC2* gene, encoding complex III core protein 2, was reported in three patients, in association with mitochondrial complex III deficiency nuclear type 5 (MC3DN5, MIM 615160), presenting with neonatal-onset metabolic acidosis, hyperammonemia and hypoglycemia. The mutation, by disrupting the hydrophobic core at the interface of the UQCRC2-containing complex, caused decreased expression or instability of core protein 2 and decreased complex III assembly and activity [143]. Various disease phenotypes associated with the mutations of the alpha 1 and other subunits of mitochondrial ATP synthase (complex V) have been reviewed in 5.1.3.

#### 5.3.3. Antioxidant Defense Protein DJ-1 (Parkinson Protein 7, PARK7)

Antioxidant defense protein *DJ-1 (Parkinson protein 7, PARK7)* was found to be a target of oxidative damage in the brains of patients with sporadic Parkinson’s disease (PD) and AD [131]. DJ-1 is a protein deglycase that repairs glyoxal- and methylglyoxal-glycated cysteinyl, arginyl and lysyl residues in proteins, reactivating their functions and preventing the formation of AGEs [144]. It protects cells against hydrogen peroxide-induced death [145] and metal toxicity [146] and is required for correct mitochondrial morphology and function, and for autophagy of dysfunctional mitochondria [147,148]. Under oxidative stress, it translocates from the cytoplasm to mitochondria and the nucleus, where it exerts a cytoprotective effect [149]. DJ-1 was detected in tau inclusions in the brains of patients with tauopathies [150]. Western blot analysis of tissue protein extracts from PD and AD brains separated by 2-D gel electrophoresis revealed increased levels of DJ-1, while mass spectrometric analysis showed that DJ-1 was irreversibly oxidized by carbonylation and methionine oxidation, suggesting the possible association of oxidative damage to DJ-1 with sporadic PD and AD [131].

Mutations of the *PARK7* gene are associated with Parkinson’s disease 7 (PARK 7, MIM 606324), an autosomal recessive, early-onset form of PD. This is a neurodegenerative disorder characterized by resting and postural tremor, bradykinesia, muscular rigidity, anxiety and psychotic episodes. PARK7 has its onset before 40 years and exhibits slow progression and good initial response to levodopa.

#### 5.3.4. Mitochondrial NADH-Ubiquinone Oxidoreductase (Respiratory Complex I)

Human NADH-ubiquinone reductase (Respiratory Complex I, EC 1.6.5.3) consists of at least 36 nuclear-encoded and seven mitochondrial-encoded subunits. Impaired catalytic activity of complex I was found in multiple tissues in PD [151]. Keeney *et al.* performed a detailed quantitative, redox and functional analysis of complex I and its subunits in enriched brain mitochondrial fractions from 10 PD patients and 12 matched controls. Using complex I-specific immunocapture antibodies and anti-DNPH antibodies, they found that complex I had in PD brains a slightly increased content of ND6 protein, a 34% lower content of 8 kDa subunit and a 47% increase of carbonyl groups in catalytic subunits. This pattern of oxidative damage could be reproduced by incubating brain mitochondria with NADH, in the presence of rotenone, but not by exposure to exogenous oxidants. The rate of NADH-driven electron transfer through complex I correlated inversely with the degree of oxidative damage. The decrease in activity of complex I in PD brain mitochondria thus correlated with misassembly and reflected the oxidation of its subunits by reactive species from an internal source under stressful conditions [132].

Isolated complex I deficiency (MIM 252010) is the most common enzymatic defect among the inherited disorders of oxidative phosphorylation and is highly genetically heterogeneous. It is associated with the mutations of 23 nuclear-encoded genes, including 16 subunits and five complex assembly genes, as well as six mitochondrial-encoded subunit genes. Its phenomics include a wide range of disorders, from lethal neonatal disease to adult-onset neurodegeneration. Clinical phenotypes include progressive leukodystrophy with macrocephaly, encephalopathy, hypertrophic cardiomyopathy, myopathy, liver disease, Leigh syndrome, LHON, and some forms of Parkinson’s disease (MIM 556500) as well.

## 6. Conclusions

Cancer and neurodegenerative diseases are both characterized by enhanced production of ROS, mainly originated from mitochondrial oxidative phosphorylation. The resulting redox imbalance is a major cause of DNA, protein, lipid damage and dysregulation of apoptotic pathways. Its deleterious effects are readily manifested also at the subcellular level, deeply affecting mitochondria. Increased ROS production in mitochondria of normal cells can induce carcinogenesis by altering mitochondrial respiration, inducing mtDNA mutations and activating several of the signaling pathways that are critical for tumor growth and maintenance. On the other hand, in cancer cells, increased ROS production can induce apoptosis and enhance cancer cell susceptibility to chemotherapy and radiation therapy [151,152]. By inducing malignant cell death, ROS might thus hinder cancer progression. Indeed, cancer therapy with pro-oxidant compounds has become a promising area of investigation [153,154].

Furthermore, ROS can induce the generation of reactive electrophilic compounds, capable of modifying mitochondrial proteins and altering their functions. The adducts of the products of lipid peroxidation (LPO) with mitochondrial proteins involved in energy metabolism, ATP production, antioxidant defense, stress responses and other vital cell functions appear to participate not only in carcinogenesis, but also in the pathogenesis of neurodegenerative diseases, such as Alzheimer’s, Huntington’s and Parkinson’s diseases. In several instances, the detection of oxidative modifications of mitochondrial proteins, in the form of newly formed 2,4-DPNH-reactive carbonyl groups, protein-bound *N*-(MDA)-lysine and HNE-protein adducts, was accompanied by the verification of their functional consequences on protein activities. In the absence of functional tests, support of the view that oxidative modification of crucial cell proteins might negatively affect mitochondrial functions and cell viability comes from a survey of human disease phenotypes associated with the mutations of the mitochondrial proteins targeted by LPO products in the context of neurodegeneration.

A number of antioxidants, including various plant-derived phenolic compounds, are under investigation as chemopreventive agents, capable of maintaining or restoring cell redox balance for the purpose of preventing cancer and neurodegeneration [155]. Notwithstanding some encouraging results of the *in vitro* experiments, the use of antioxidants for treating neurodegenerative diseases has been tested repeatedly in clinical trials, without convincing results [156,157]. The identification and characterization of definite molecular targets of oxidative modification in mitochondria, involved in the pathogenesis of cancer and neurodegeneration, in addition to providing a rationale for the mitochondrial dysfunctions that have been documented in these disease conditions, may foster a deeper understanding of the mechanisms by which oxidative stress participates in their pathogenesis, thus inspiring more rational therapeutic strategies for the control of cellular redox imbalance and its undesirable effects. In perspective, the identification of such targets may pave the way to the design and experimentation of specific transcriptional modulators, protein stabilizers, allosteric modifiers and enzymatic cofactors of the proteins in point. In due time, these advances may provide us not only with the conceptual and analytical means that are required for more detailed molecular diagnoses, but also with active compounds to be used in targeted molecular therapies that may be tailored to the individual needs of patients, as the emerging concepts of personalized medicine demand.

## Figures and Tables

**Table 1:** Percentage content of different phospholipid classes in mammalian mitochondria [46].

Phospholipids	Percentage Content	
	OMM	IMM
Phosphatidylcholine	54	40
Phosphatidylethanolamine	29	34
Phosphatidylinositol	13	5
Phosphatidyserine	2	3
Cardiolipin	<1	18
Others	<1	0

OMM, outer mitochondrial membrane; IMM, inner mitochondrial membrane.

**Table 2:** Monounsaturated and polyunsaturated fatty acid content in phospholipid classed of mitochondria isolated from rat liver hepatoma.

Tissues/Cells	Phophatidyl^−^ Choline		Phophatidyl^−^ Ethanolamine		Phosphatldylserine^+^ Phosphatidylinositol		Cardiolipn	
	MUFA	PUFA	MUFA	PUFA	MUFA	PUFA	MUFA	PUFA
Normal liver	11.41	37.11	7.08	43.11	6.84	30.18	18.04	55.46
Nodules	23.62	27.24	19.06	34.85	14.44	24.89	25.37	36.27
Hepatoma	29.68	25.68	22.57	37.78	22.12	25.64	28.54	23.72
AH-130 Hepatoma	25.33	26.28	18.54	47.21	17.83	28.23	22.17	28.75

MUFA, sum of monounsaturated fatty acids; PUFA, sum of polyunsaturated fatty acids [68].

**Table 3:** HNE effects on mitochondrial proteins in cancer models.

Cancer Model	Protein Targets	Mitochondrial Function	HNE Effect	Reference
PC12 pheochromocytoma cell line	cytochrome c oxidase aconitase	respiratory enzymes	inhibition	[81]
Kidney cancers	HNE-mitochondrial protein adducts			[101]
Skin carcinogenesis	HNE-mitochondrial protein adducts	-	-	[102]
Breast cancer cells	sirtuin 3 (SIRT3) HNE-SIRT3 adducts	NAD^+^-dependent deacetylase	inhibition	[103]
HeLa cervical adenocarcinoma cell line	thioredoxin reductase (TrxR)	Trx reduction	inhibition	[104]
RKO colorectal carcinoma cell line	-	cytochrome c release	induction	[105]
RAW 264.7 mouse monocytic/macrophagic leukemic cell line	-	cytochrome c release	induction	[106]

**Table 4:** Adducts of aldehydes derived from lipid peroxidation with mitochondrial proteins in neurodegenerative diseases, in relation with clinical progression.

Protein	AD Stage	Function	Reference
*HNE-modified proteins*			
Aconitate hydratase, mitochondrial (Aconitase 2, ACO2)	LAD	energy metabolism, mitochondrial function	[121]
ATP synthase (complex V) alpha subunit 1 (ATP5A1)	PAD, MCI, EAD, LAD	energy metabolism, ATP production	[109,110,121,128]
Translation elongation factor Tu (EF-Tu, TUFM)	MCI	protein synthesis	[109]
Malate dehydrogenase 2, mitochondrial (MDH2)	EAD	energy metabolism, gluconeogenesis	[110]
Mn Superoxide dysmutase, mitochondrial (SOD2)	EAD, LAD	antioxidant defense	[110,121]
*MDA-modified proteins*			
Ubiquinol-cytochrome c reductase (complex III) core protein 1 (UQCRC1)	LAD	electron transport, ATP production	[116]
ATP synthase (complex V) beta subunit (ATP5B)	LAD	energy metabolism, ATP production	[116]
60-kDa Heat shock protein (HSPD1, HSP60)	LAD	stress response	[116]
Glutamate dehydrogenase 1, mitochondrial (GDH1)	LAD	energy metabolism	[116]
*Proteins with increased content of DPNH-reactive groups*			
Aconitate hydratase, mitochondrial (Aconitase 2, ACO2)	HD	energy metabolism, mitochondrial function	[129]
Citrate synthase, mitochondrial (CS)	HD	energy metabolism	[130]
Creatine kinase B, ubiquitous mitochondrial (CKMT1A)	HD	ATP production	[130]
Ubiquinol-cytochrome c reductase (complex III) core protein 2 (UQCRC2)	HD	electron transport, ATP production	[130]
ATP synthase (complex V) alpha subunit 1 (ATP5A1)	HD	energy metabolism, ATP production	[130]
DJ-1 (Parkinson protein 7, PARK7)	PD, AD	antioxidant defense	[131]
NADH-ubiquinone oxidoreductase (complex I)	PD	electron transport, ATP production	[132]

PAD, preclinical Alzheimer’s disease (Braak I/II stage); MCI, amnestic mild cognitive impairment (follows in time PAD and precedes EAD, during AD progression); EAD, early-stage Alzheimer’s disease; LAD, late-stage Alzheimer’s disease; HD, Huntington’s disease; PD, Parkinson’s disease.
